# Systematic Review of Prevalence Studies and Familial Aggregation in Vestibular Migraine

**DOI:** 10.3389/fgene.2020.00954

**Published:** 2020-08-31

**Authors:** Ana Paz-Tamayo, Patricia Perez-Carpena, Jose A. Lopez-Escamez

**Affiliations:** ^1^Division of Otolaryngology, Department of Surgery, Universidad de Granada, Granada, Spain; ^2^Department of Otolaryngology, Instituto de Investigación Biosanitaria ibs.GRANADA, Hospital Universitario San Cecilio, Granada, Spain; ^3^Otology & Neurotology Group CTS495, Department of Genomic Medicine, GENYO - Centre for Genomics and Oncological Research – Pfizer/University of Granada/Junta de Andalucía, PTS, Granada, Spain; ^4^Department of Otolaryngology, Instituto de Investigación Biosanitaria ibs.GRANADA, Hospital Universitario Virgen de las Nieves, Granada, Spain

**Keywords:** vestibular migraine, heritability, prevalence, genetics, vestibular disorders, epidemiology

## Abstract

**Background:** Vestibular migraine (VM) is complex disorder consisting of episodes of migraine and vertigo with an estimated prevalence of 1–3%. As migraine, it is considered that VM has genetic predisposition; however, evidence to support a genetic contribution has not been critically appraised.

**Objective:** The aim of this systematic review is to assess available evidence in scientific publications to determine the role of inheritance in VM.

**Methods:** After performing the quality assessment of the retrieved records, 31 studies were included (24 epidemiological reports and 7 genetic association studies in families or case-control in candidate genes). We gathered data about prevalence of VM in different populations and in families, and also about the genetic findings reported. In addition, other variables were considered to assess the heritability of VM, such as the ancestry, the age of onset or the familial history of vertigo and migraine.

**Results:** The estimated prevalence of VM was different between black (3.13%), white (2.64%) and Asian (1.07%) ethnicities. The reported prevalence of VM in migraine patients is higher in European countries (21%) than in Asian countries (10%). Moreover, the prevalence of the migraine-vertigo association in families is 4–10 times higher than the prevalence reported in the general population (sibling recurrence risk ratio λ_s_ = 4.31–10.42). We also found that the age of onset is lower in patients with simultaneous onset of symptoms and in those who have familial history for migraine and/or vertigo, suggesting anticipation. Although some genetic studies have reported few allelic variants associated to MV, replication studies are needed to validate these results.

**Conclusions:** The available evidence to support heritability in VM is limited. Variability in prevalence depending on ethnicity and geographic location suggests a combined genetic and environmental contribution to VM. However, the familial aggregation observed in VM support genetic and shared familial environmental effects that remarks the necessity of twins and adoptees-based epidemiological studies to estimate its heritability.

## Introduction

Migraine is a complex multifactorial disorder characterized by headache attacks associated with a constellation of neurological symptoms. In approximately one-third of patients, headaches are preceded by transient focal sensorial symptoms, so-called auras, involving visual and hearing systems. Genetic factors contribute to the clinical spectrum of migraine and multiple common variants have been associated with migraine with and without aura (Sutherland and Griffiths, 2017; de Boer et al., 2019). The diagnostic criteria of migraine were standardized according to the International Headache Society (Headache Classification Committee of the International Headache Society, 2018). Epidemiological evidence supports that familial aggregation with an early disease onset, particularly for the aura subtype, indicating a higher genetic susceptibility in migraine with aura (Russell et al., 1996; Ulrich et al., 1999; Mulder et al., 2003; Stewart et al., 2006).

Vestibular migraine (VM) consist of a subgroup of patients with migraine where the sensorial symptoms involve the vestibular system. VM is characterized by recurrent episodes of vertigo, a current or past history of migraine and simultaneous occurrence of both symptoms during crisis (Li et al., 2019). VM is one of the most common causes of recurrent vertigo. Prevalence studies have described MV as a frequent disease (0.9–2.7%), with variations in the frequency according to the population of study (Neuhauser et al., 2006; von Brevern et al., 2007; Formeister et al., 2018). However, VM is underdiagnosed, due to variability and overlap of symptoms with other causes of vertigo and to normal neurological examination and neuroimaging (Li et al., 2019).

The diagnostic criteria for VM were jointly developed by the Barany Society and the International Headache Society in 2012 (Lempert et al., 2012), and they differentiate probable and definite VM. Criteria for definite VM were included in the appendix of the 3rd edition of the International Classification of Headache Disorders (ICHD-III), one step forward to consider VM as an independent entity (Headache Classification Committee of the International Headache Society, 2018).

Migraine is a complex and multifactorial disorder with a large genetic component (Sutherland and Griffiths, 2017). Therefore, VM could also present a genetic predisposition (Knezevic et al., 2018; Rainero et al., 2019).

Familial clustering in VM has been occasionally observed, supporting the hypothesis of a genetic contribution to VM (Espinosa-Sanchez and Lopez-Escamez, 2015). Some studies reported families with an autosomal dominant inheritance with a moderate to high penetrance; however, no causative mutations have been found (Kim et al., 1998; von Brevern et al., 2006). Moreover, some loci segregating VM have been identified by linkage analysis in some few families with affected individuals in 22q12 (Lee et al., 2006) and 5q35 (Bahmad et al., 2009). These results suggest polygenic inheritance for VM; however, evidence to support a genetic contribution has not been critically appraised, and more research in this area is needed.

The aim of this systematic review is to assess available evidence in scientific publications to determine the role of inheritance in VM. So, we gathered information about the prevalence of VM in different populations, including family studies and twin studies, and we analyzed the quality of reported findings in genetic studies.

## Materials and Methods

### Study Design

This review has followed the Preferred Reported Items for Systematic Reviews and Meta-Analyses (PRISMA) guidelines (Moher et al., 2009) (Supplementary Table 1).

According to the methodology for systematic reviews, the issues related to the PICO question are listed below and the studies have been selected according to the following characteristics:

- Participants: Patients diagnosed with VM.
- Intervention: Measurement of prevalence of the disease, estimation of familial aggregation, estimation of concordance in monozygotic/dizygotic twins, measurement of the presence of certain genes or loci, measurement of other demographic characteristics (age of onset of symptoms, familial history of migraine, vertigo or the vertigo-headache association).
- Control: Controlled and uncontrolled studies.
- Main results: Variation in the prevalence of the disease among different populations, families and twins compared to the general population.
- Secondary results: Reported associations with certain genes or loci; variation in the prevalence of other demographic characteristics (age of onset or family history).
- Study design: Case-control studies, family studies, twin concordance studies, cross-sectional studies and case series.

### Search Strategy

The article search was performed on May 23, 2020 in the PubMed and Scopus databases, with the following combination of keywords: (“vestibular migraine” OR “migrainous vertigo” OR “migraine associated vertigo” OR “migraine related vertigo”) AND (“epidemiology” OR “prevalence” OR “inheritance” OR “heritage” OR “heritability” OR “genes” OR “genetics” OR “families” OR “familial” OR “twins”). The search was limited to articles published in the last 25 years. Additional records identified through the list of references or other sources were also included.

### Exclusion Criteria

Records that met the following characteristics were excluded from the review:

- Animal studies.
- Studies in child population.
- Articles published in languages other than English or Spanish.

### Data Collected

Two independent reviewers (APT, PPC) analyzed the scientific papers that met the selection criteria. Each article was reviewed to extract the relevant data for the purpose of this work. The main data that were extracted from the studies were those referring to indicators of heritability, since the main objective of this review is to assess whether VM is a disease that has a genetic contribution. These heritability criteria in multifactorial disorders are estimated by comparing the variation of the prevalence of the disease among population with different ethnic background and by familial aggregation studies calculating the sibling recurrence risk ratio (λ_s_) using the Falconer formula (Wickramaratne and Hodge, 2001).

The following information was also collected from descriptive and genetic studies: first author and year of publication, country, study design, main objective, sample size, MV diagnostic criteria, ancestry, sex of patients with VM, mean age of patients with VM and mean age of VM onset. From descriptive studies information on VM prevalence, target population, and family history of MV was also extracted.

### Data Synthesis

Information on the prevalence of VM have been collected in epidemiological studies. Mean values and standard deviation have been calculated for the age of onset of migraine and vertigo. Prevalence of VM in families was estimated from different studies and pooled to calculate the recurrence risk among siblings (λ_s)_. Statistical analyses were performed using Microsoft Excel and SPPS software.

### Quality Assessment

The quality of each study has been assessed through the Cochrane Collaboration Tool (Higgins et al., 2019) and the risk of bias was summarized in Supplementary Table 2. Furthermore, the quality of genetic studies has also been evaluated according to the criteria defined to assess genetic studies in quantitative traits with extreme phenotype (Amanat et al., 2020).

## Results

**Figure 1:**
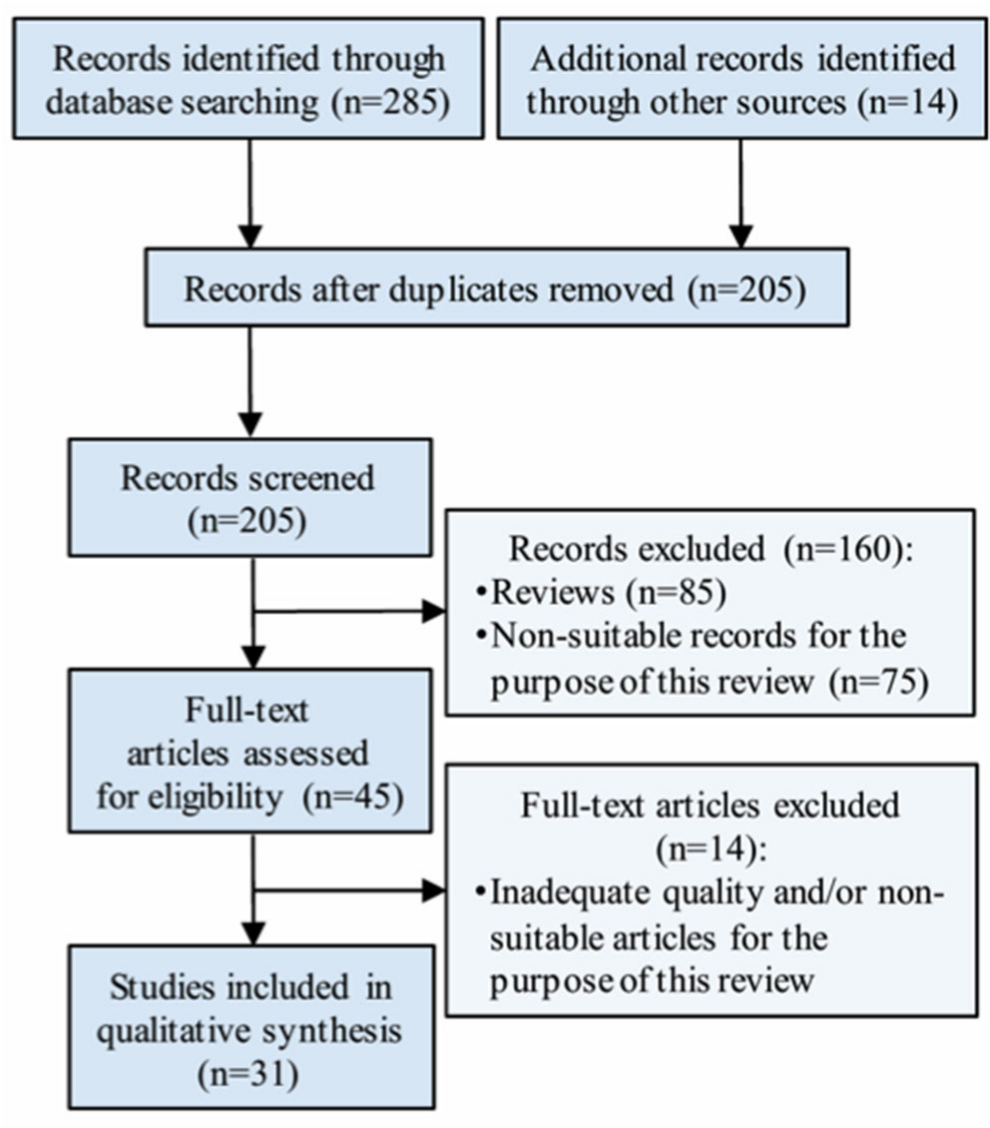
Selection of studies.

Thirty-one studies with a total sample size of 41,127 individuals were finally included in this revision, according to the eligibility criteria. A flowchart detailing the selection of studies is included in Figure 1.

### Cross-Sectional Studies and Case Series

Twenty-four descriptive epidemiological studies were included (16 cross-sectional studies, 5 case series, and 3 familial studies) (Supplementary Table 3). These studies estimated the prevalence of VM in different populations, geographical areas or families and reported clinical and demographic features of this disease. Five studies were population-based, while the rest were hospital-based.

Only 5 studies mentioned the ancestry and 3 of them included data about the prevalence of VM. An earlier onset of the disease was found in families when they were compared to non-familiar VM patients.

A female preponderance was observed in VM, although four studies did not report this information. Nineteen studies reported the mean age of patients with VM, however, only 11 described the age at VM onset. Thus, familial history of VM patients was detailed in seven studies.

### Genetic Studies

Seven genetic studies were included (3 case-control studies and 4 genetic linkage analysis in families) (Table 1). The main purpose of these studies was to find mutations or loci in families associated with the disease. One of the case-control studies reported a significant association of the allelic variant rs770963777 in the *HTR6* gene with VM in a small cohort of Han Chinese descendants. Two of the studies reported significant associations of VM with certain genome regions: a locus in chromosome 5q35, and the PROGINS variant of the *PRG* gene, respectively. Another study found significant association between chromosome 22q12 and benign recurrent vertigo, which was a common diagnosis established for patients with migraine and episodic vertigo before the VM diagnostic criteria were established.

**Table 1:** Summary of the six genetic studies in patients with vestibular migraine.

**References**	**Country**	**Study design**	**Main objective**	**Sample size**	**Main findings**	**Diagnostic criteria (VM)**	**Ancestry**	**Gender of VM patients (% women)**	**Mean age of VM patients (years)**	**Mean age of VM onset (years)**
Wu et al. (2020)	China	Case-control	To investigate the association of rs770963777 in HTR6 gene with VM	- 92 VM - 100 controls	Significant association of VM with rs770963777(C/T)	Barany/IHS	Han Chinese	52.2%	44.2 ± 9.3	Not available
Peddareddygari et al. (2019)	USA	Genome-wide linkage analysis in one family using microsatellite markers	To test whether vertigo and motion sickness are inherited through different susceptibility genes than migraine	29	Non-significant results	- ICHD-I: migraine - *ad hoc* clinical criteria: vertigo and motion sickness	Not available	90%	Not available	10.5 ± 4
Bahmad et al. (2009)	USA/Brazil	Genome-wide linkage analysis in one family using microsatellite markers	To map the genetic locus for familial VM and to define the progression of the disease in one family	23	Significant association of VM with a region of chromosome 5q35	Neuhauser et al.	Not available	50%	60.7 ± 20.6	- 12± 4.7 (migraine) - 39.2 ± 6.2 (vertigo)
Lee et al. (2008)	USA	Genetic linkage analysis in one family	To analyze phenotypic and genetic features of a family with VM in order to assess its inheritance pattern.	46	- Non-significant results - Non-significant association with chromosome 11q in most affected women	- ICHD-I: migraine - *ad hoc* criteria: VM	Not available	87.5%	47.6 ± 15.4	- 14.7 ± 6 (migraine) - 36.2 ± 9.1 (vertigo) - 13.5 ± 9.2 (simultaneous onset of both symptoms)
Lee et al. (2007)	USA	Case-control	To test the association of female hormonal genes (PGR and ESR1) with VM	- 150 MV - 145 controls	Significant association of VM with PROGINS variant of progesterone receptor	- IHS: migraine - *ad hoc* clinical criteria: vertigo	Caucasian	83.4%	Not available	Not available
von Brevern et al. (2006)	USA/Germany	Case-control	To test whether mutations in CACNA1A, ATP1A2, SCN1A and CACNB4 confer susceptibility to VM	- 14 MV - 46 controls	Non-significant results	Neuhauser et al. (modified)	German, Turkish, Bosnian	64.3%	50 ± 11.1	- 21.3 ± 7.5 (migraine) - 37.9 ± 14.4 (vertigo) - 35.3 ± 13.4 (simultaneous onset of both symptoms)
Lee et al. (2006)	USA	Genome-wide linkage analysis in families	To genetically define BRV and its association with migraine.	257	- 31.2% prevalence of migraine and BRV association - Significant association of chromosome 22q12 with BRV	- IHS criteria for migraine - *ad hoc* clinical criteria for vertigo	Not available	84.4%	Not available	Not available

*VM, Vestibular Migraine; ICHD, International Classification of Headache Disorders; IHS, International Headache Society; BRV, Benign Recurrent Vertigo*.

Three studies reported the ancestry, specifically Han Chinese in one of the studies, Caucasian in another one and German, Bosnian and Turkish in the last one. Female preponderance was also observed in all genetic studies.

### Prevalence of VM According to the Geographic Area or Ancestry

Two population-based studies estimated the prevalence of VM in the general population, ranging from 0.89 to 2.70% in Germany and US, respectively. However, most studies were hospital-based and the authors estimated the prevalence of VM in migraine patients and in outpatient clinics, showing some differences. Five studies used the Barany Society diagnostic criteria for VM, and showed a prevalence of VM ranging from 4.3% in Belgium to 22% in Egypt (Table 2).

**Table 2:** Prevalence of vestibular migraine depending on geographic area and target population (outpatients clinics or hospital-based studies).

**References**	**Continent**	**Country**	**Target population**	**Number of patients**	**Diagnostic criteria**	**Prevalence of VM**
Power et al. (2018)	Oceania	Australia	Outpatients in a balance disorders clinic	90	Barany/IHS	41% definite and probable VM
Yollu et al. (2017)	Europe	Turkey	Patients with migraine	100	Barany/IHS	21% definite VM
Hazzaa and El Mowafy (2016)	Africa	Egypt	Outpatients in a dizziness clinic	446	Barany/IHS	22% definite VM
Akdal et al. (2015)	Europe	Turkey	Patients with migraine	871	- ICHD-II for migraine. - *ad hoc* clinical criteria for vestibular symptoms	- 62% vertigo - 76% vestibular symptoms (vertigo and motion sickness)
Akdal et al. (2013)	Europe	Turkey	Patients with migraine	1,880	- ICHD-II for migraine. - *ad hoc* clinical criteria for vestibular symptoms	20.3% vestibular symptoms (vertigo and/or dizziness and/or motion sickness)
Jay-du Preez and van Papendorp (2011)	Africa	South Africa	Patients visiting a general practitioner	717	Neuhauser et al.	1.67% (definite and probable VM)
Uneri (2004)	Europe	Turkey	Patients with BPPV	476	ICHD-I for migraine	54.8% migraine
Van Ombergen et al. (2015)	Europe	Belgium	Patients attending an ORL clinic	407	Barany/IHS	- 4.3% definite VM - 5.6% probable VM
Vuković et al. (2007)	Europe	Croatia	Patients with migraine	327	Neuhauser et al.	23.2% definite VM
Neuhauser et al. (2001)	Europe	Germany	- Patients with migraine - Patients attending a dizziness clinic	- 200 with migraine - 200 attending a dizziness clinic	Neuhauser et al.	- 9% definite VM in patients with migraine - 7% definite VM in patients attending a dizziness clinic
Cho et al. (2016)	Asia	South Korea	Patients with migraine	631	Barany/IHS	- 10.3% definite VM - 2,5% probable VM
Tungvachirakul et al. (2014)	Asia	Thailand	Patients attending a neurotology clinic	167	Neuhauser et al.	34.7% definite VM

*VM, Vestibular Migraine; IHS, International Headache Society; ICHD, International Classification of Headache Disorders; BPPV, Benign Paroxysmal Positional Vertigo*.

One population-based study performed in US described that 13.9% of VM patients were black, while 79.8% of VM patients were white, which results in a significant difference. After the analysis of the results from this population-based survey, we calculated the prevalence of VM for each ethnicity, resulting in a VM prevalence of 3.13% in African descendants, 2.64% in Europeans and 1.07% in Asian descendent population.

### Familial Aggregation

Three studies reported that the migraine-vertigo association was more frequent in siblings of patients with VM than in the general population.

On the other hand, some cross-sectional and case series studies described a familial history of migraine, vertigo or both among VM patients. So, a familial history of migraine was reported in 70% of VM patients and 21.4% of VM patients had a familial history of VM.

The sibling recurrence risk ratio (λ_s_) for the migraine-vertigo association was calculated by comparing the prevalence of this association in families with that prevalence in the general population, to assess the familial aggregation for VM (Table 3). Our results also showed a moderate familial aggregation (λ_s_ = 4.31–10.42).

**Table 3:** Prevalence of migraine-vertigo association in families and sibling recurrence risk (λ_s_).

**References**	**Sample size**	**Number of siblings with migraine and vertigo**	**Total number of siblings**	**Prevalence of migraine and vertigo in siblings (%)**	**λ_**s**_**
Peddareddygari et al. (2019)^*^	29	4	15	26.67	8.33
Bahmad et al. (2009)	23	4	12	33.33	10.42
Cha et al. (2008)	69	4	29	13.79	4.31
Lee et al. (2008)	46	2	14	14.29	4.46
Lee et al. (2006)	257	13	57	22.81	7.13
Oh et al. (2001)	287	20	68	29.41	9.19
Oliveira et al. (1997)	19	5	18	27.78	8.68
Total	730	52	213	24.41	7.63

* *Studies based on Barany Society diagnostic criteria for VM*.

### Age of VM Onset

We compared the age of VM onset in different studies, particularly, the age of migraine onset, the age of vertigo onset and the simultaneous onset of vertigo and migraine, including a brief analysis on sex distribution (Table 4). So, we found that the mean age of onset in patients with simultaneous presentation of vertigo and migraine was 22.7 ± 10.4 years; however, for patients with a metachronic presentation of symptoms, the mean age of onset for migraine was 24 ± 8.9 years, and 35.6 ± 12.4 year for vertigo.

**Table 4:** Sex distribution and age of onset (mean ± standard deviation) of migraine, vertigo, and patients with simultaneous onset of both symptoms.

**References**	**Number of patients with VM and non- simultaneous onset of migraine and vertigo (sex distribution, F/M)**	**Age of migraine onset (years)**	**Age of vertigo onset (years)**	**Number of patients with VM and simultaneous onset of migraine and vertigo**	**Simultaneous onset for migraine and vertigo (years)**
^*^Beh et al. (2019)	129	–	44.3 ± 13.7	–	–
^*^Peddareddygari et al. (2019)	10 (9/1)	–	10.5 ± 4	–	–
Teggi et al. (2018)	260	21.8 ± 9	37.4 ± 13.1	19	19.8 ± 2.1
Martínez et al. (2017)	14 (14/0)	16.3 ± 8.2	31.7 ± 11.8	27	24 ± 12
^*^Cohen et al. (2011)	147	30.7	38.7	–	–
Bahmad et al. (2009)	6 (2/4)	12 ± 4.7	39.2 ± 6.2	–	–
Lee et al. (2008)	6 (5/1)	14.7 ± 6	36.2 ± 9.1	2	13.5 ± 9.2
^*^Vuković et al. (2007)	169 (migraine with vestibular symptoms)	–	25.3	–	–
von Brevern et al. (2006)	11 (6/5)	21.3 ± 7.5	37.9 ± 14.4	3	35.3 ± 13.4
^*^Neuhauser et al. (2001)	33	22 ± 11	35 ± 14	–	–
^*^Oh et al. (2001)	20	–	34.15 ± 16.9	–	–
Oliveira et al. (1997)	8 (5/3)	15.5 ± 10.1	–	–	–
Female total	41	15, 2+/-7, 4	33, 7+/- 15, 9	21	24, 8+/- 12, 8
Male total	14	19, 9+/-10, 1	37, 5+/-14, 3	11	23, 6+/-13, 3
Total	813	24 ± 8.9	35.6 ± 12.4	51	22.7 ± 10.4

*VM, Vestibular Migraine*.

* *These studies do not report data on simultaneous age of onset; therefore, they were included in the “patients with non-simultaneous onset” category*.

To sum up, we observed that 6 studies recorded data about audiological symptoms in VM. The prevalence and distribution of these audiological symptoms in VM is shown in Table 5.

**Table 5:** Prevalence of audiological symptoms in patients with VM.

**References**	***N***	**Audiological symptoms reported**	**Number of patients with otological symptoms**	**Prevalence**
Teggi et al. (2018)	252	Tinnitus (during vertigo attacks)	27	0.11
		Ear fullness (during vertigo attacks)	22	0.09
		Hearing loss (during vertigo attacks)	10	0.04
Yollu et al. (2017)	21	Sensorineural hearing loss (SNHL) according to average hearing threshold	2	0.09
		SNHL acc. to low frequency	6	0.28
		SNHL acc. to high frequency	11	0.52
Van Ombergen et al. (2015)	65	Tinnitus	35	0.54
		Decreased hearing	3	0.05
		Hearing loss	5	0.25
Cha et al. (2008) (families study)	20	Migraine and MD symptoms (migraine/MD)	19	0.13
Lee et al. (2007)	150	Migraine and MD symptoms (migraine/MD)	19	0.13
Neuhauser et al. (2006)	33	Cochlear symptoms (during vertigo attacks)	12	0.36
		Tinnitus (during vertigo attacks)	5	0.15
		Aural fullness (during vertigo attacks)	5	0.15
		Hearing loss (during vertigo attacks)	3	0.09
Total	541	Hearing loss	46	0.085
	350	Tinnitus	67	0.191

## Discussion

The association between migraine and vertigo has been known for a long time; however, it has not been considered as an independent entity until the last decade (56). The current diagnostic criteria were published in 2012 (Lempert et al., 2012), therefore, only 8 of the studies included in this revision applied these criteria for the diagnosis of VM. The criteria proposed by Neuhauser et al. (2001) were reported in 8 of the studies, and in the rest of the publications, the authors used ICHD criteria for migraine and *ad-hoc* clinical criteria for vertigo. One of the family studies did not report any diagnostic criteria, but later this family was reported as familial VM.

There are several evidence to support heritability in complex traits such as vestibular disorders: (a) differences in the prevalence of the condition according to the ethnic background; (b) familial aggregation with early onset and anticipation; and (c) high concordance of the phenotype in monozygotic twins and adoptees with biological parents when they are compared with dizygotic twins or adoptive parents, respectively (Gallego-Martinez et al., 2018).

The population-based study from Formeister et al. (2018) showed a higher prevalence of VM in African American or Europeans than in Asian descendent population, suggesting a possible genetic contribution. It should be interesting to compare the prevalence of VM in East Asia in a large cohort with the Asian American descendent to assess the environmental effect on VM.

Moreover, the prevalence of VM in migraine patients seems to be higher in European countries (23% in Croatia, 21% in Turkey) (Vuković et al., 2007; Yollu et al., 2017) than in Asian countries (10% in South Korea) (Cho et al., 2016). However, the diagnosis of VM in neurotology clinics shows a great variation ranging from a 7% reported in Germany (Neuhauser et al., 2001), 22% in Egypt (Hazzaa and El Mowafy, 2016) to 41% in Australia (Power et al., 2018). These data suggest differences in the diagnostic criteria used rather than ethnic differences in VM and evidence that population-based studies are needed to estimate the prevalence of VM.

Familial aggregation is usually reported in rare diseases, and suggests a combined effect of genetic and environmental factors (Requena et al., 2014a). This type of studies compares the prevalence of a disease in individuals of the same generation within a family with the prevalence of that disease in general population. In order to do this, the sibling recurrence risk ratio (λ_s_) is calculated. That proportion allows us to know how many times the disease is more frequent between the siblings of the affected individual, as compared with the general population. Our systematic review show that familial studies (Oliveira et al., 1997; Oh et al., 2001; Lee et al., 2006, 2008; Cha et al., 2008; Bahmad et al., 2009; Peddareddygari et al., 2019) showed a higher prevalence of the migraine-vertigo association than the observed prevalence of that association in the general population (3.2%) (Neuhauser et al., 2001), and also higher than the 2.7% prevalence of VM in general population (Formeister et al., 2018). We calculated λ_s_ for the migraine-vertigo association, which resulted in a risk ranging between 4–10 times higher than in general population.

Most of the studies included in this review reported information about age of onset of migraine and vertigo. Some of them described a significantly lower age of onset in patients with VM, as compared with controls affected either by migraine or vertigo (Vuković et al., 2007; Akdal et al., 2013). By comparing the age of VM onset in several studies, we found that there was an earlier onset of symptoms in those patients with simultaneous presentation of migraine and vertigo, as compared with those with metachronic onset of symptoms.

Early and simultaneous onset of symptoms observed in familial VM suggests genetic anticipation, a phenomenon of progression of severity of an inherited disorder in successive generations frequently found in neurological disorders and associated with expansion of nucleotide repeats (Carpenter, 1994). Teggi et al. (2018) reported a significantly lower age of migraine onset in patients with a familial history of migraine or VM, as compared with those without it. These results were also reported in some of the included familial studies (Lee et al., 2008; Bahmad et al., 2009; Peddareddygari et al., 2019), in which age of migraine onset seems to be lower than in non-familial cases.

The genetic association studies retrieved have reported allelic variants in the *HTR6* and *PRG* genes associated with VM (Lee et al., 2007; Wu et al., 2020). Replication studies are needed to validate these results.

The genetic contribution to vestibular diseases, including VM is largely unknown (Requena et al., 2014b; Gallego-Martinez et al., 2018). The application of high throughput sequencing technologies in multiplex families with deep phenotyping will target candidate genes and clarify disease mechanisms and facilitate the genetic diagnosis of VM in clinical practice (Di Resta and Ferrari, 2018; Prodan Žitnik et al., 2018).

### Limitations of This Study

Most of the genetic studies included in this revision are based on linkage analysis of multicase families. The majority of them were published more than a decade ago and were performed before the development of high-throughput DNA massively parallel sequencing technology. Only two of them found 2 loci associated with VM, but none of them found the causal mutations.

The current diagnostic criteria for VM were published in 2012, so previous studies applied different probably broader criteria. This variability in the diagnostic criteria is a selection bias that might influence the estimated prevalence and therefore, could limit the comparability between studies. Moreover, the number of studies analyzing VM heritability could be considered low, so few of them share the same variables, resulting in a limited comparability.

### Future Perspectives

This systematic review provides some evidences to support a genetic contribution in VM, including familial aggregation. There is a need to perform twins and adoptees studies to estimate heritability in VM. Therefore, whole genome sequencing studies in multicase families are needed to find genetic variants conferring susceptibility to this disease.

## Conclusions

1. Clinical studies seem to report differences in the prevalence of VM according to the ethnic origin and country.
2. Family aggregation studies show a higher prevalence of migraine and vertigo in families compared to general population, with a moderate risk of recurrence among siblings and possible anticipation.
3. Since there are no twins studies published, there is a need to perform such studies to estimate heritability in VM.

## Author Contributions

JL-E conceived the study design and develop the scientific arguments. AP-T and PP-C performed literature search, quality assessment of the studies, and interpretation of data. All authors drafted the manuscript and revised the final version.

## Conflict of Interest

The authors declare that the research was conducted in the absence of any commercial or financial relationships that could be construed as a potential conflict of interest.

## References

[B1] Akdal G. Baykan B. Ertaş M. Zarifoğlu M. Karli N. Saip S.. (2015). Population-based study of vestibular symptoms in migraineurs. Acta Otolariyngol. 135, 435–439. 10.3109/00016489.2014.969382 25662067

[B2] Akdal G. Ozge A. Ergör G. (2013). The prevalence of vestibular symptoms in migraine or tension-type headache. J. Vestib. Res. 23, 101–106. 10.3233/VES-130477 23788138

[B3] Amanat S. Requena T. Lopez-Escamez J. A. (2020). A systematic review of extreme phenotype strategies to search for rare variants in genetic studies of complex disorders. Genes (in press). 32854191 10.3390/genes11090987 PMC7564972

[B4] Bahmad F. DePalma S. R. Merchant S. N. Bezerra R. L. Oliveira C. A. Seidman C. E.. (2009). Locus for familial migrainous vertigo disease maps to chromosome 5q35. Ann. Otol. Rhinol. Laryngol. 118, 670–676. 10.1177/000348940911800912 19810609 PMC2767209

[B5] Beh S. Masrour S. Smith S. Friedman D. (2019). The spectrum of vestibular migraine: clinical features, triggers and examination findings. Headache 59, 727–740. 10.1111/head.13484 30737783

[B6] Carpenter N. J. (1994). Genetic anticipation. expanding tandem repeats. Neurol. Clin. 12, 683–697. 10.1016/S0733-8619(18)30071-9 7845337

[B7] Cha Y. H. Kane M. J. Baloh R. W. (2008). Familial clustering of migraine, episodic vertigo, Ménière's disease. Otol. Neurotol. 29, 93–96. 10.1097/mao.0b013e31815c2abb 18046258 PMC2820370

[B8] Cho S. J. Kim B. K. Kim B. S. Kim J. M. Kim S. K. Moon H. S.. (2016). Vestibular migraine in multicenter neurology clinics according to the appendix criteria in the third beta edition of the international classification of headache disorders. Cephalalgia 36, 454–462. 10.1177/0333102415597890 26224714

[B9] Cohen J. Bigal M. Newman L. (2011). Migraine and vestibular symptoms - identifying clinical features that predict vestibular migraine. Headache 51, 1393–1397. 10.1111/j.1526-4610.2011.01934.x 21649658

[B10] de Boer I. van den Maagdenberg A. M. J. M. Terwindt G. M. (2019). Advance in genetics of migraine. Curr. Opin. Neurol. 32, 413–421. 10.1097/WCO.0000000000000687 30883436 PMC6522206

[B11] Di Resta C. Ferrari M. (2018). Next generation sequencing: from research area to clinical practice. EJIFCC 29, 215–220. 30479607 PMC6247137

[B12] Espinosa-Sanchez J. M. Lopez-Escamez J. A. (2015). New insights into pathophysiology of vestibular migraine. Front. Neurol. 6:12. 10.3389/fneur.2015.00012 25705201 PMC4319397

[B13] Formeister E. J. Rizk H. G. Kohn M. A. Sharon J. D. (2018). The Epidemiology of vestibular migraine: a population-based survey study. Otol. Neurotol. 39, 1037–1044. 10.1097/MAO.0000000000001900 30020261

[B14] Gallego-Martinez A. Espinosa-Sanchez J. M. Lopez-Escamez J. A. (2018). Genetic contribution to vestibular diseases. J. Neurol. 265(Suppl. 1), 29–34. 10.1007/s00415-018-8842-7 29582143

[B15] Hazzaa N. El Mowafy S. S. (2016). Clinical features of vestibular migraine in Egypt. Egypt. J. Ear Nose Throat Allied Sci. 17, 17–21. 10.1016/j.ejenta.2015.12.002

[B16] Headache Classification Committee of the International Headache Society (2018). (IHS) The international classification of headache disorders, 3rd edition. Cephalalgia. 38, 1–211. 10.1177/0333102417738202 29368949

[B17] Higgins J. Thomas J. Chandler J. Cumpston M. Li T. Page M. (2019). Cochrane Handbook for Systematic Reviews of Interventions version 6.0 Cochrane. Chichester, UK: John Wiley & Sons.

[B18] Jay-du Preez T. van Papendorp D. (2011). Migraine-associated vertigo and dizziness as presenting complaint in a private general medical practice. South Afr. Family Pract. 53, 165–169. 10.1080/20786204.2011.10874079

[B19] Kim J. S. Yue Q. Jen J. C. Nelson S. F. Baloh R. W. (1998). Familial migraine with vertigo: no mutations found in CACNA1A. Am. J. Med. Genet. 79, 148–151. 10.1002/(SICI)1096-8628(19980901)79:2<148::AID-AJMG11>3.0.CO;2-J 9741473

[B20] Knezevic N. N. Tverdohleb T. Knezevic I. Candido K. D. (2018). The role of genetic polymorphisms in chronic pain patients. Int. J. Mol. Sci. 19:1707. 10.3390/ijms19061707 29890676 PMC6032204

[B21] Lee H. Jen J. C. Cha Y. H. Nelson S. F. Baloh R. W. (2008). Phenotypic and genetic analysis of a large family with migraine-associated vertigo. Headache 48, 1460–1467. 10.1111/j.1526-4610.2007.01002.x 18081823 PMC2846425

[B22] Lee H. Jen J. C. Wang H. Chen Z. Mamsa H. Sabatti C.. (2006). A genome-wide linkage scan of familial benign recurrent vertigo: linkage to 22q12 with evidence of heterogeneity. Hum. Mol. Genet. 15, 251–258. 10.1093/hmg/ddi441 16330481

[B23] Lee H. Sininger L. Jen J. C. Cha Y. H. Baloh R. W. Nelson S. F. (2007). Association of progesterone receptor with migraine-associated vertigo. Neurogenetics 8, 195–200. 10.1007/s10048-007-0091-3 17609999

[B24] Lempert T. Olesen J. Furman J. Waterston J. Seemungal B. Carey J.. (2012). Vestibular migraine: diagnostic criteria. J. Vestib. Res. 22, 167–172. 10.3233/VES-2012-0453 23142830

[B25] Li V. McArdle H. Trip S. A. (2019). Vestibular migraine. BMJ 366:l4213. 10.1136/bmj.l4213 31270067

[B26] Martínez E. Ruiz-Piñero M. de Lera M. Barón J. Pedraza M. Guerrero-Peral A. (2017). Clinical characteristics of vestibular migraine: considerations in a series of 41 patients. Rev. Neurol. 64, 1–6. 10.33588/rn.6401.2016164 28000906

[B27] Moher D. Liberati A. Tetzlaff J. Altman D. G. Group P. (2009). Preferred reporting items for systematic reviews and meta-analyses: the PRISMA statement. J. Clin. Epidemiol. 62, 1006–1012. 10.1016/j.jclinepi.2009.06.005 19631508

[B28] Mulder E. J. Van Baal C. Gaist D. Kallela M. Kaprio J. Svensson D. A.. (2003). Genetic and environmental influences on migraine: a twin study across six countries. Twin Res. 6, 422–431. 10.1375/136905203770326420 14624726

[B29] Neuhauser H. Leopold M. von Brevern M. Arnold G. Lempert T. (2001). The interrelations of migraine, vertigo, migrainous vertigo. Neurology 56, 436–441. 10.1212/WNL.56.4.436 11222783

[B30] Neuhauser H. K. Radtke A. von Brevern M. Feldmann M. Lezius F. Ziese T.. (2006). Migrainous vertigo: prevalence and impact on quality of life. Neurology 67, 1028–1033. 10.1212/01.wnl.0000237539.09942.06 17000973

[B31] Oh A. K. Lee H. Jen J. C. Corona S. Jacobson K. M. Baloh R. W. (2001). Familial benign recurrent vertigo. Am. J. Med. Genet. 100, 287–291. 10.1002/ajmg.1294 11343320

[B32] Oliveira C. A. Bezerra R. L. Araújo M. F. Almeida V. F. Messias C. I. (1997). Menière's syndrome and migraine: incidence in one family. Ann. Otol. Rhinol. Laryngol. 106, 823–829. 10.1177/000348949710601004 9342978

[B33] Peddareddygari L. R. Kramer P. D. Hanna P. A. Levenstien M. A. Grewal R. P. (2019). Genetic analysis of a large family with migraine, vertigo, motion sickness. Can. J. Neurol. Sci. 46, 512–517. 10.1017/cjn.2019.64 31258098

[B34] Power L. Shute W. McOwan B. Murray K. Szmulewicz D. (2018). Clinical characteristics and treatment choice in vestibular migraine. J. Clin. Neurosci. 52, 50–53. 10.1016/j.jocn.2018.02.020 29550250

[B35] Prodan Žitnik I. Cerne D. Mancini I Simi L. Pazzagli M. Di Resta C.. (2018). Personalized laboratory medicine: a patient-centered future approach. Clin. Chem. Lab. Med. 56, 1981–1991. 10.1515/cclm-2018-0181 29990304

[B36] Rainero I. Vacca A. Govone F. Gai A. Pinessi L. Rubino E. (2019). Migraine: genetic variants and clinical phenotypes. Curr. Med. Chem. 26, 6207–6221. 10.2174/0929867325666180719120215 30027842

[B37] Requena T. Espinosa-Sanchez J. Lopez-Escamez J. (2014b). Genetics of dizziness: cerebellar and vestibular disorders. Curr. Opin. Neurol. 27, 98–104. 10.1097/WCO.0000000000000053 24275721

[B38] Requena T. Espinosa-Sanchez J. M. Cabrera S. Trinidad G. Soto-Varela A. Santos-Perez S.. (2014a). Familial clustering and genetic heterogeneity in Meniere's disease. Clin. Genet. 85, 245–252. 10.1111/cge.12150 23521103

[B39] Russell M. B. Iselius L. Olesen J. (1996). Migraine without aura and migraine with aura are inherited disorders. Cephalalgia 16, 305–309. 10.1046/j.1468-2982.1996.1605305.x 8869764

[B40] Stewart W. F. Bigal M. E. Kolodner K. Dowson A. Liberman J. N. Lipton R. B. (2006). Familial risk of migraine: variation by proband age at onset and headache severity. Neurology 66, 344–348. 10.1212/01.wnl.0000196640.71600.00 16476932

[B41] Sutherland H. G. Griffiths L. R. (2017). Genetics of migraine: insights into the molecular basis of migraine disorders. Headache 57, 537–569. 10.1111/head.13053 28271496

[B42] Teggi R. Colombo B. Albera R. Asprella Libonati G. Balzanelli C. Batuecas Caletrio A.. (2018). Clinical features, familial history, and migraine precursors in patients with definite vestibular migraine: the VM-phenotypes projects. Headache 58, 534–544. 10.1111/head.13240 29205326

[B43] Tungvachirakul V. Lisnichuk H. O'Leary S. (2014). Epidemiology of vestibular vertigo in a neuro-otology clinic population in Thailand. J. Laryngol. Otol. 128, S31–S38. 10.1017/S0022215113003484 24548658

[B44] Ulrich V. Gervil M. Kyvik K. O. Olesen J. Russell M. B. (1999). The inheritance of migraine with aura estimated by means of structural equation modelling. J. Med. Genet. 36, 225–227. 10204850 PMC1734315

[B45] Uneri A. (2004). Migraine and benign paroxysmal positional vertigo: an outcome study of 476 patients. Ear Nose Throat J. 83, 814–815. 10.1177/014556130408301211 15724736

[B46] Van Ombergen A. Van Rompaey V. Van De Heyning P. Wuyts F. (2015). Vestibular migraine in an otolaryngology clinic: prevalence, associated symptoms, and prophylactic medication effectiveness. Otol. Neurotol. 36, 133–138. 10.1097/MAO.0000000000000596 25251304

[B47] von Brevern M. Radtke A. Lezius F. Feldmann M. Ziese T. Lempert T.. (2007). Epidemiology of benign paroxysmal positional vertigo: a population based study. J. Neurol. Neurosurg. Psychiatr. 78, 710–715. 10.1136/jnnp.2006.100420 17135456 PMC2117684

[B48] von Brevern M. Ta N. Shankar A. Wiste A. Siegel A. Radtke A.. (2006). Migrainous vertigo: mutation analysis of the candidate genes CACNA1A, ATP1A2, SCN1A, and CACNB4. Headache 46, 1136–1141. 10.1111/j.1526-4610.2006.00504.x 16866717

[B49] Vuković V. Plavec D. Galinović I Lovrencić-Huzjan A. Budisi,ć M Demarin V. (2007). Prevalence of vertigo, dizziness, and migrainous vertigo in patients with migraine. Headache 47, 1427–1435. 10.1111/j.1526-4610.2007.00939.x 18052952

[B50] Wickramaratne P. Hodge S. (2001). Estimation of sibling recurrence-risk ratio under single ascertainment in two-child families. Am. J. Hum. Genet. 68, 807–812. 10.1086/318784 11179030 PMC1274495

[B51] Wu X. Qiu F. Wang Z. Liu B. Qi X. (2020). Correlation of 5-HTR6 gene polymorphism with vestibular migraine. J. Clin. Lab. Anal. 34:e23042. 10.1002/jcla.23042 31587366 PMC7031542

[B52] Yollu U. Uluduz D. U. Yilmaz M. Yener H. M. Akil F. Kuzu B.. (2017). Vestibular migraine screening in a migraine-diagnosed patient population, and assessment of vestibulocochlear function. Clin. Otolaryngol. 42, 225–233. 10.1111/coa.12699 27385658

