# Supplementary material for: Systematic Review of Prevalence Studies and Familial Aggregation in Vestibular Migraine
Source: Front Genet. 2020 Aug 31;11:954. doi: 10.3389/fgene.2020.00954 (PMC7489493; doi:10.3389/fgene.2020.00954)
Supplement: Supplementary file 1 [file Data_Sheet_1.docx]

Supplementary Material

Supplementary table 1. Preferred Reported Items for Systematic Reviews and Meta-Analyses (PRISMA)

| **Sección/topic** | **#** | **Checklist item** | **Reported on page #** |
| --- | --- | --- | --- |
| **TITLE** |  |  |  |
| Title | 1 | Identify the report as a systematic review, meta-analysis, or both. | Title page |
| **ABSTRACT** |  |  |  |
| Structured summary | 2 | Provide a structured summary including, as applicable: background, objectives; data sources; study eligibility criteria, participants, and interventions; study appraisal and synthesis methods; results; limitations; conclusions and implications of key findings; systematic review registration number. | 2 |
| **INTRODUCTION** |  |  |  |
| Rationale | 3 | Describe the rationale for the review in the context of what is already known. | 3 |
| Objectives | 4 | Provide an explicit statement of questions being addressed with reference to participant, interventions, comparisons, outcomes, and study design (PICOS). | 3 |
| **METHODS** |  |  |  |
| Protocol and registration | 5 | Indicate if a review protocol exists, if and where it can be accessed (e.g., Web address), and, if available, provide registration information including registration number. | 3 |
| Eligibility criteria | 6 | Specify study characteristics (e.g., Web address), and, if available, provide registration information including registration number. | 4 |
| Information sources | 7 | Describe all information sources (e.g., databases with dates of coverage, contact with study authors to identify additional studies) in the search and date last searched. | 4 |
| Search | 8 | Present full electronic search strategy for at least one database, including any limits used, such that it could be repeated. | 4 |
| Study selection | 9 | State the process for selecting studies (i.e., screening, eligibility, included in systematic review, and, if applicable, included in the meta-analysis). | 4 |
| Data collection process | 10 | Describe method of data extraction from reports (e.g., piloted forms, independently, in duplicate) and any processes for obtaining and confirming data from investigators. | 4 |
| Data items | 11 | List and define all variables for which data were sought (e.g., PICOS, funding sources) and any assumptions and simplifications made. | 4 |
| Risk of bias in individual studies | 12 | Describe methods used for assessing risk of bias of individual studies (including specification of whether this was done at the study or outcome level), and how this information is to be used in any data synthesis. | 5 |
| Summary measures | 13 | State the principal summary measures (e.g., risk ratio, difference in means). | 4-5 |
| Synthesis of results | 14 | Describe the methods of handling data and combining results of studies, if done, including measures of consistency (e.g., I^2^) for each meta-analysis. | 4-5 |
| Risk of bias across studies | 15 | Specify any assessment of risk of bias that may affect the cumulative evidence (e.g., publication bias, selective reporting within studies). | 5 |
| Additional analyses | 16 | Describe methods of additional analyses (e.g., sensitivity or subgroup analyses, meta-regression), if done, indicating which were pre-specified. | 4-5 |
| **RESULTS** |  |  |  |
| Study selection | 17 | Give numbers of studies screened, assessed for eligibility, and included in the review, with reasons for exclusions at each stage, ideally with a flow diagram. | 5 |
| Study characteristics | 18 | For each study, present characteristics for which data were extracted (e.g., study size, PICOS, follow-up period) and provide the citations. | 5 |
| Risk of bias within studies | 19 | Present data on risk of bias of each study and, if available, any outcome level assessment (see item 12). | 5 |
| Results of individual studies | 20 | For all outcomes considered (benefits or harms), present, for each study: (a) simple summary data for each intervention group (b) effect estimates and confidence intervals, ideally with a forest plot. | NA |
| Synthesis of results | 21 | Present results of each meta-analysis done, including confidence intervals and measures of consistency. | NA |
| Risk of bias across studies | 22 | Present results of any assessment of risk of bias across studies (see Item 15). | 5 |
| Additional analysis | 23 | Give results of additional analyses, if done (e.g., sensitivity or subgroup analyses, meta-regression [see Item 16]). | 5-6 |
| **DISCUSSION** |  |  |  |
| Summary of evidence | 24 | Summarize the main findings including the strength of evidence for each main outcome; consider their relevance to key groups (e.g., healthcare providers, users, and policy makers). | 6-7 |
| Limitations | 25 | Discuss limitations at study and outcome level (e.g., risk of bias), and at review-level (e.g., incomplete retrieval of identified research, reporting bias). | 7 |
| Conclusions | 26 | General interpretation of the results in the context of other evidence, and implications for future research. | 8 |
| **FUNDING** |  |  |  |
| Funding | 27 | Describe sources of funding for the systematic review and other support (e.g., supply of data); role of funders for the systematic review. | 8 |

NA: Not Applicable

**Supplementary table 2.** Summary of risk of bias

| **Reference** | **Sample size** | **Study design** | **Selection bias** | **Detection bias** | **Attrition bias** |
| --- | --- | --- | --- | --- | --- |
| Wu *et al.,* 2020 | 192 | Genetic study (case-control) | High | Low | Low |
| Beh *et al*., 2019 | 131 | Case series | High | NA | High |
| Peddareddygari *et al*., 2019 | 29 | Genetic linkage analysis in one family | High | NA | High |
| Formeister *et al*., 2018 | 21781 | Cross-sectional | Low | Low | High |
| Teggi *et al.*, 2018 | 252 | Cross-sectional | Low | Low | Low |
| Teggi *et al.*, 2018 | 279 | Cross-sectional | Low | Low | Low |
| Power *et al.,* 2018 | 90 | Case series | High | NA | Low |
| Yollu *et al.*, 2017 | 100 | Cross-sectional | High | Low | Low |
| Martínez *et al.*, 2017 | 41 | Case series | High | NA | Low |
| Cho *et al.*, 2016 | 1414 | Cross-sectional | High | Unclear | Low |
| Hazzaa *et al.,* 2016 | 446 | Cross-sectional | High | NA | Low |
| Van Ombergen *et al.*, 2015 | 407 | Cross-sectional | High | Unclear | Unclear |
| Akdal *et al.*, 2015 | 871 | Cross-sectional | Low | High | Low |
| Tungvachirakul *et al.*, 2014 | 167 | Cross-sectional | High | Low | Low |
| Akdal *et al.*, 2013 | 5111 | Cross-sectional | High | High | Low |
| Hsu *et al.*, 2011 | 1436 | Cross-sectional | High | High | High |
| Cohen *et al.*, 2011 | 147 | Case series | High | High | High |
| Jay-du Preez *et al.,* 2011 | 717 | Cross-sectional | High | Low | Low |
| Salhofer *et al.*, 2010 | 116 | Cross-sectional | High | High | High |
| Bahmad *et al.*, 2009 | 23 | Genetic linkage analysis in one family | High | Low | Low |
| Cha *et al.*, 2008 | 69 | Familial study | High | High | Low |
| Lee *et al.*, 2008 | 46 | Genetic linkage analysis in one family | High | NA | High |
| Vuković *et al.*, 2007 | 651 | Cross-sectional | High | Low | Low |
| Lee *et al.*, 2007 | 295 | Genetic study (case-control) | High | Low | Low |
| Neuhauser *et al.*, 2006 | 4869 | Cross-sectional | Low | Low | High |
| Von Brevern *et al.*, 2006 | 60 | Genetic study (case-control) | Unclear | Low | Low |
| Lee *et al.*, 2006 | 257 | Genetic linkage analysis in one family | High | Low | High |
| Uneri, 2004 | 476 | Case series | High | Unclear | Low |
| Neuhauser *et al.*, 2001 | 600 | Cross-sectional | High | Low | High |
| Oh *et al.*, 2001 | 287 | Familial study | High | Low | High |
| Oliveira *et al.*, 1997 | 19 | Familial study | High | Low | High |

NA: Not Applicable

**Supplementary table 3.** Summary of 21 descriptive studies

| **Reference** | **Country** | **Study design** | **Main objective** | **Sample size** | **Indicators of heritability** | **Prevalence of VM** | **Target population** | **Diagnostic criteria for VM** | **Ancestry** | **Gender (VM) (% women)** | **Mean age (VM) (years)** | **Mean age of VM onset (years)** | **Familial history** |
| --- | --- | --- | --- | --- | --- | --- | --- | --- | --- | --- | --- | --- | --- |
| Beh *et al.*, 2019 | USA | Case series | To assess clinical characteristics of VM | 131 | - | - | Hospital-based: patients with definite VM | Barany/IHS | Not available | 80.2 | Not available | 44.3± 13.7 (vestibular symptoms) | - 50.8% (migraine)  - 28.1% (vestibular symptoms) |
| Formeister *et al.*, 2018 | USA | Cross-sectional | To describe the epidemiology of VM in the USA and characterize the sociodemographic and clinical attributes of VM patients | 21781 | - Significantly higher proportion of black individuals in the group of VM as compared with all respondents  - Significantly lower mean age in VM patients as compared with all respondents | 2.7% (one-year prevalence) | Population-based: adult US population | Barany/HIS (aprox) | For VM patients: 79.8% White; 13.9% Black; 1.8% Asia; 0.8% Indian; 3.1% Multiple race | 75.8 | 40.9 | Not available | Not available |
| *Teggi *et al.*, 2018 | Italy and Spain | Cross-sectional | To assess the clinical characteristics, familial history and migraine precursors in patients with definite VM | 252 | -Significantly lower age of onset in patients with simultaneous onset of migraine and vertigo as compared with those without simultaneous onset  - Significantly lower age of migraine onset in patients with familial history for migraine or VM as compared with those without familial history | - | Hospital-based: patients with definite VM | Barany/IHS | Caucasian | 84.9 | 45.8± 13.6 | - 23± 9 (migraine)  - 38± 13 (vertigo)  - 20± 2 (simultaneous onset of migraine and vertigo) | - 70.2% (migraine)  - 66.3% (vertigo)  - 21.4% (MV) |
| *Teggi *et al.*, 2018 | Italy and Spain | Cross-sectional | To assess the clinical characteristics, familial history and migraine precursors in patients with definite VM | 279 | Significantly lower age of onset in patients with simultaneous onset of migraine and vertigo as compared with those without simultaneous onset | - | Hospital-based: patients with definite VM | Barany/IHS | Caucasian | 84.2 | 45.8± 13.8 | - 21.8± 9 (migraine)  - 37.4± 13.1 (vertigo)  - 19.8± 2.1 (simultaneous onset of migraine and vertigo) | 67.4% (migraine) |
| Power *et al.,* 2018 | Australia | Case series | To describe the characteristics of VM and its management | 90 | - | 41% (definite and probable VM) | Hospital-based: patients attending a balance disorders clinic | Barany/IHS | Not available | 80 | 50 (17-84) | Not available | 22% (migraine) |
| Yollu *et al.*, 2017 | Turkey | Cross-sectional | To estimate the prevalence of VM in migraine patients and to identify | 100 | - | 21% (definite VM) | Hospital-based: patients with migraine | Barany/IHS | Not available | 95.2 | 36± 10.9 | Not available | Not available |
| Martínez *et al.*, 2017 | Spain | Case series | To analyze demographic and clinical characteristics of VM patients | 41 | - | - | Hospital-based: patients with definite VM | Barany/IHS | Not available | 73.2 | 31.8± 13.3 | - 16.3± 8.2 (migraine)  - 31.7± 11.8 (vertigo)  - 24± 12 (simultaneous onset of migraine and vertigo) | 26.8% (migraine) |
| Cho *et al.*, 2016 | South Korea | Cross-sectional | To assess the prevalence and clinical characteristics of VM | 1414 | The mean age, proportion of females and age at headache onset, did not differ significantly between patients with VM and those with non-VM. | - 4.6% definite VM (headache patients)  - 10.3% definite VM (migraine patients) | Hospital-based:  - Patients with headache  - Patients with migraine | Barany/IHS | Korean | 78.5 | 40.3 | 30 (21 – 40.5) (migraine) (median) | Not available |
| Hazzaa *et al.,* 2016 | Egypt | Cross-sectional | To determine the prevalence of vestibular migraine in a dizziness clinic and to describe its clinical profile | 446 | - | 22% (definite VM) | Hospital-based: patients consulting to a dizziness clinic | Barany/IHS | Not available | 57 | 33.8± 11.3 | Not available | 16% (migraine) |
| Van Ombergen *et al.*, 2015 | Belgium | Cross-sectional | To assess the prevalence of VM in patients consulting to an ORL clinic | 407 | - | - 4.3% definite VM  - 5.6% probable VM | Hospital-based: patients consulting to an ORL clinic | Barany/IHS | Not available | 64.7 | 51± 13.9 | Not available | Not available |
| Akdal *et al.*, 2015 | Turkey | Cross-sectional | To assess vestibular symptoms in patients with migraine | 871 | - | - 62% (vertigo)  - 76% (vestibular symptoms) | Population-based: patients with migraine | ICHD-II criteria for migraine Ad hoc clinical criteria for vestibular symptoms | Not available | Not available | 37± 11 | Not available | Not available |
| Tungvachirakul *et al.*, 2014 | Thailand | Cross-sectional | To assess the epidemiology of VM | 167 | - | 34.7% definite VM | Hospital-based: patients consulting to a neurotology clinic | Neuhauser *et al.* | Not available | 82.8 | Not available | Not available | Not available |
| Akdal *et al.*, 2013 | Turkey | Cross-sectional | To assess the frequency of vestibular symptoms in patients consulting to a headache clinic | 5111 | The group of migraine patients with vestibular symptoms is significantly younger than the group of migraine patients without vestibular symptoms | 20.3% of migraine patients have vestibular symptoms | Hospital-based: patients with headache | - ICHD-II criteria for migraine  - Ad hoc clinical criteria for vestibular symptoms | Not available | 89 | 41.5± 12.7 | Not available | Not available |
| Hsu *et al.*, 2011 | Taiwan | Cross-sectional | To estimate the prevalence of VM in mid-life women during the menopausal transition | 1436 | - | 5% (one-year prevalence) | Community-based: mid-life women during menopausal transition | Neuhauser *et al.* (modified) | Han Chinese | - | 45.6± 4 | Not available | Not available |
| Cohen *et al.*, 2011 | USA | Case series | To define clinical and demographic characteristics of VM | 147 | - | - | Hospital-based: VM patients | - ICHD-II criteria for migraine  - Ad hoc clinical criteria for VM | Not available | 68 | 45 | - 30.7 (migraine)  - 38.7 (vestibular symptoms) | Not available |
| Jay-du Preez *et al.,* 2011 | South Africa | Cross-sectional | To investigate the prevalence of migrainous vertigo and migraine-associated dizziness as presenting complaints | 717 | - | 1.67% (definite and probable VM) | Hospital-based: patients visiting a general practitioner | Neuhauser *et al.* | Not available | 75 | 34 (14-50) | Not available | Not available |
| Salhofer *et al.*, 2010 | Austria | Cross-sectional | To assess the prevalence and characteristics of VM by means of a diary | 116 | - | 15.5% (one-month prevalence) | Population-based: patients with migraine | Neuhauser *et al.* | Not available | Not available | Not available | Not available | Not available |
| Cha *et al.*, 2008 | USA | Familial study | To evaluate the association between migraine, episodic vertigo, and Ménière’s disease in families. | 69 | Higher prevalence of episodic vertigo and migraine in families as compared with the population | - 48% (migraine)  - 32.4% (vertigo)  - 22% (migraine and vertigo, MD not included) | Families with MD and/or vertigo and/or migraine | - ICHD-II criteria for migraine  - AAO-HNS 1995 criteria for MD  - Ad hoc clinical criteria for vertigo | Not available | 80 (patients with migraine and vertigo, MD excluded) | 43.5± 11.7 (only probands) | Not available | - |
| Vuković *et al.*, 2007 | Croatia | Cross-sectional | To assess the prevalence of vertigo and dizziness in patients with migraine and to establish the prevalence of VM | - 327 migraine  - 324 controls | Significantly lower age of vertigo onset in patients with migraine as compared with controls | 23.2% (definite VM) | Hospital-based: patients with migraine | Neuhauser *et al.* | Not available | 88.4 (patients with migraine) | 39.9± 12.2 (patients with migraine) | 25.3 (vertigo) | Not available |
| Neuhauser *et al.*, 2006 | Germany | Cross-sectional | To estimate the prevalence of VM in the general population in Germany | 4869 | - | - 0.89% (one-year prevalence) definite VM  - 0.98% (lifetime prevalence) definite VM | Population-based: adult Germany general population | Neuhauser *et al.* | Not available | 83 | 43± 15 | 23 (median) | Not available |
| Uneri, 2004 | Turkey | Case series | To estimate the association between migraine and BPPV and to investigate a genetic propensity | 476 | Higher prevalence of familial history of migraine and vertigo in patients with BPPV as compared with controls | 54.8% prevalence of migraine in patients with BPPV | Hospital-based: patients with BPPV | ICHD-I criteria for migraine | Not available | 70.4 (BPPV) | 41.6± 6.7 (BPPV) | Not available | - 58.4% (migraña)  - 44.9% (episodic vertigo) |
| Neuhauser *et al.*, 2001 | Germany | Cross-sectional | To assess the prevalence of VM in patients with migraine and in patients with vertigo | - 200 migraine  - 200 dizziness clinic  - 200 controls | - | - 7% lifetime prevalence of definite VM in patients with dizziness  - 9% lifetime prevalence of definite VM in patients with migraine | Hospital-based:  - Patients with dizziness  - Patients with migraine | Neuhauser *et al.* | Not available | 81.8 | 46.4± 11.2 | - 22± 11 (migraña)  - 35± 14 (vertigo) | Not available |
| Oh *et al.*, 2001 | USA | Familial study | To assess the clinical features and mode of inheritance of the BRV and migraine association | 287 | Higher prevalence of the migraine-vertigo association in families as compared with general population | 32.4% prevalence of the migraine-vertigo association in families | Families with BRV | - ICHD-I criteria for migraine  - Ad hoc clinical criteria for vertigo | Not available | 80.4 | 44.8± 11.9 (only probands) | 34.15± 16.9 (vertigo) | - |
| Oliveira *et al.*, 1997 | Brazil | Familial study | To test the possibility of a genetic determinant in idiopathic MD as well as a possible association between migraine and MD | 19 | Higher prevalence of the migraine-vertigo association in families as compared with general population | - 64.4% prevalence of affected patients  - 22.2% prevalence of the migraine-vertigo association | Families with MD | Not available | Not available | 60 | 49.2± 15.6 | 17.7± 12.7 (migraine) | - |

VM: Vestibular Migraine; IHS: International Headache Society; ICHD: International Classification of Headache Disorders; BPPV: Bening Paroxysmal Positional Vertigo; MD: Ménière’s Disease; BRV: Benign Recurrent Vertigo. *Both studies from Teggi *et al.* based their results on the same group of patients.
